# A preliminary pan-European assessment of pollution loads from urban runoff

**DOI:** 10.1016/j.envres.2020.109129

**Published:** 2020-03

**Authors:** A. Pistocchi

**Affiliations:** European Commission, Joint Research Centre (JRC), Ispra, Italy

**Keywords:** Urban runoff, Diffuse pollution, Event mean concentration, Large scale assessment

## Abstract

Acknowledging the difficulty of modelling pollution conveyed by urban runoff, this contribution presents a first pan-European quantification of loads from this diffuse source. We estimate annual loads of 5-days biochemical oxygen demand (BOD_5_), nitrogen (N), phosphorus (P) and total suspended solids (TSS) using a simple event mean concentration (EMC) model initially proposed by Heaney et al., 1976. On a European scale, this yields discharges corresponding to untreated wastewater of about 31 million population equivalents (PE) for BOD_5_, about 18.5 million PE for N and P and about 280 million for TSS. These represent 51% of the pollution coming from treated wastewater for BOD_5_, 15% for N and P and 461% for TSS. Although the model applied for the assessment was developed more than 40 years ago, the results are consistent with those obtained using more recent parameterizations, except for a tendency to underestimate P loads. Although lack of data on pollution from urban runoff makes model verification impossible, and the uncertainty on EMC models is known to be very high, urban runoff emerges as a significant source of pollution, and should be properly addressed as such. Reducing runoff volume from urban areas through improved water retention is not only key to pollution control, but also a no-regret option thanks to its co-benefits, especially when incorporated at early stages of planning and design.

## 1 Introduction

With the progress of urban wastewater treatment, the wash-off of urban impervious surfaces represents an increasing percentage of the load of contaminants potentially discharged to the European stream network. It is therefore important to quantify this load in order to inform future policy making on urban water management.

Pollution associated to urban runoff is unfortunately difficult to quantify. The most sophisticated models generally rely on the simulation of pollutant build-up on urban surfaces during dry weather, followed by wash-off during storm events. Models of this kind have been calibrated and used in specific cases, but have a high demand for site-specific data. “*Simulation of urban runoff is a very inexact science if it can even be called such. Very large uncertainties arise both in the representation of the physical, chemical and biological processes and in the acquisition of data and parameters for model algorithms.* […] *It is unrealistic to assume that* [these can achieve] *enough accuracy to determine a priori the amount of pollutants on the surface at the beginning of the storm. Equally naive is the idea that empirical washoff equations truly represent the complex hydrodynamic (and chemical and biological) processes that occur while overland flow moves in random patterns over the land surface*.” (Rossman and Huber, 2016, p. 42). When the wealth of data required for sophisticated models is not available, i.e. in almost all cases, use of this type of models is generally expected to yield questionable advantages over much simpler and cheaper approaches (*ibid.*). A practical alternative for the quantification of urban runoff loads is the use of “event mean concentrations” (EMCs) to represent the pollutant content of urban runoff. In turn, urban runoff volume is expected to be well explained by the volume of rainfall on impervious urban surfaces. The mass of the pollutant discharged to receiving water bodies from an urban surface is then computed simply as the product of a pollutant's EMC and urban runoff volume. The concentration of a pollutant during a storm event varies depending on a number of processes, and significant variability occurs also from event to event. One can only rely on this approach if the adopted EMC is truly representative of concentrations typically found in runoff, and the uncertainty of quantifications based on EMC is on a par with the variability of EMCs themselves. This paper presents a first quantification of urban runoff loads for selected pollutants at the European scale, based the parameterization of EMC proposed by Heaney et al. (1976), with the goal of understanding their importance also compared to wastewater loads. It should be stressed that urban runoff is very often mixed with wastewater contributions whenever the sewer system is combined. In Europe, although wastewater treatment systems receive runoff during smaller storms, combined sewer overflows may occur with a certain frequency during larger storms. The analysis presented here neglects the additional pollution conveyed by overflows, as well as the abatement of loads from runoff conveyed to wastewater treatment through combined sewers. This enables appraising the loads associated with urban runoff alone, in order to understand their relevance in comparison with other pollution sources.

The results of the analysis cannot be validated in the substantial absence of data; however, they are shown to be comparable with more recently suggested EMC, lending credibility to this preliminary estimation. In the following sections we illustrate the approach and data used in the quantification, present and discuss the results aggregated by European regions and countries. Based on the results, we draw some recommendations for policy.

## 2 Methods

We assume that all runoff coming from a given type of land use carry a constant equivalent concentration equal to the event mean concentration (EMC). The load of the generic ith pollutant carried by urban runoff [M] [T]^−1^ is represented as:

(1) $$L_{i}=\overset{n}{\underset{j=1}{\sum }}EMC_{i,j}R_{j}$$

where EMC_i_ is the event mean concentration of the pollutant [M] [L]^−3^, and R_j_ is the annual runoff [L]^3^ [T]^−1^ from urban areas with a land use of type j. In the approach proposed by Heaney et al. (1976) (see also Pistocchi, 2014; Pistocchi et al., 2014), the runoff volume is estimated as:

(2) $$R_{j}=f_{j}P_{j}$$

where f is the runoff coefficient (f = 1 for industrial and commercial land uses, f = 0.142 for land uses not related to population such as cemeteries, parks or schools, and a function of population density D (persons/acre) in residential land uses: f = 0.142 + 0.218 D^0.54^. The values of EMC suggested by Heaney et al. (1976), for 5-days biochemical oxygen demand (BOD_5_), total nitrogen (N), total phosphorus (P), and total suspended solids (TSS) for the different land use types and the case of separate sewers are provided in Table 1 (Heaney et al., 1976, also suggest EMC values for combined sewers, which are not of interest in this exercise). The Authors propose to account for the effect of street cleaning by multiplying EMC by a factor $s=\text{min}\left(1,\;\frac{N}{20}\right)$, where N is the interval (days) between two operations of sweeping. In this work, we set s = 1. We use population density data from the GEOSTAT dataset (European Commission, 2011), and urban land use data from the Corine Land Cover 2012 (CLC2012) dataset (https://land.copernicus.eu/pan-european/corine-land-cover/clc-2012). CLC2012 is reclassified into “residential”, “commercial or industrial” and “other” land use in order to apply the EMC values in Table 1. Precipitation data are taken from the CRU gridded climatology (New et al., 2002) as in Pistocchi et al., 2014).

**Table 1:** EMC for selected pollutants for different land use types, according to Heaney et al. (1976). Values in lb/(acre in), and correspondence between CLC land cover classes and land use types for attribution of EMC values.

Land use	CLC classes	BOD_5_	Suspended solids	Volatile solids	Total phosphate (as PO_4_)	Total nitrogen (as N)
Residential	111, 112	0.799	16.3	9.45	0.0336	0.131
Commercial	121 to 124	3.2	22.2	14.0	0.0757	0.296
Industrial	131 to 133	1.21	29.1	14.3	0.0705	0.277
Other	141, 142	0.113	2.70	2.6	0.00994	0.0605

## 3 Results and discussion

**Fig. 1:**
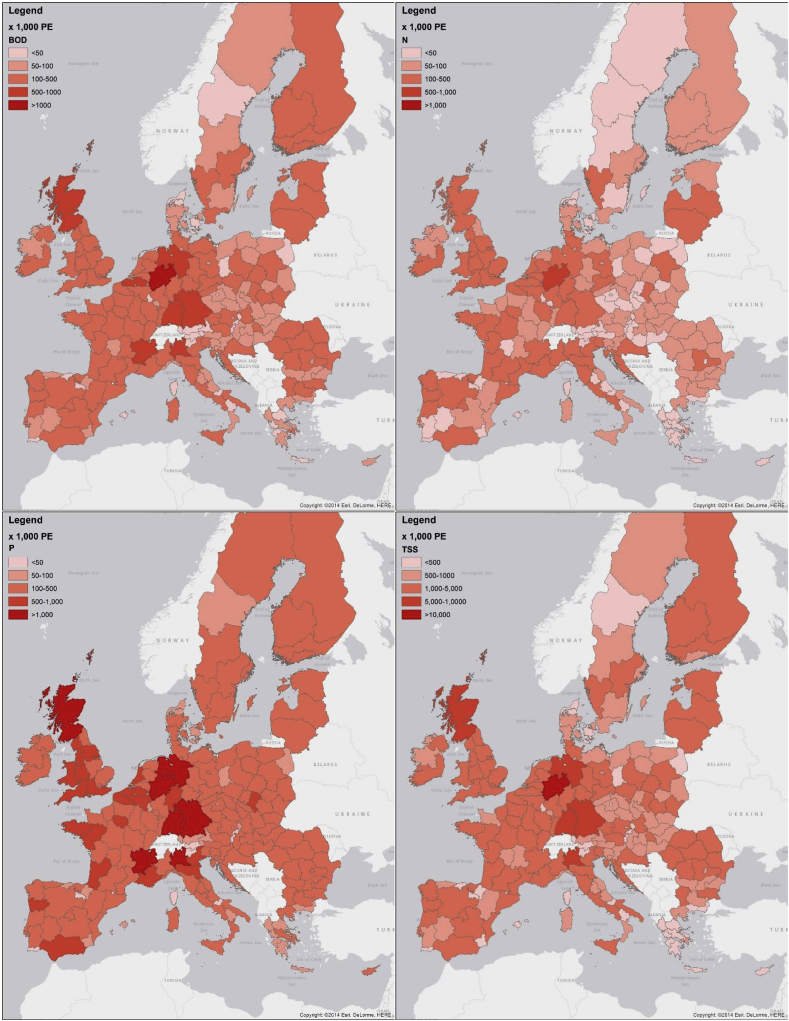
Estimated loads of BOD, total P, total N and total suspended sediments (TSS) from urban runoff for European regions, expressed as population equivalents (PE).

Fig. 1 shows maps of loads of BOD_5_, TSS, P (as PO_4_) and N estimated for the various European regions. These are expressed as “population equivalents” (PE) where 1 PE conventionally corresponds to 60 g of BOD_5_, 12 g of N, 1 g of P and 80 g of TSS daily. The results reflect the patterns of annual precipitation, impervious area and population in a rather intuitive way. Depending on the region, urban runoff accounts for loads that represent between 100,000 and 1,000,000 PE or more, and only in a few regions with very limited urban areas these numbers fall below 50,000 PE. The PE sum up over the EU to 31 millions for BOD_5_, 18.5 millions for N and P, and 280 millions for TSS.

**Fig. 2:**
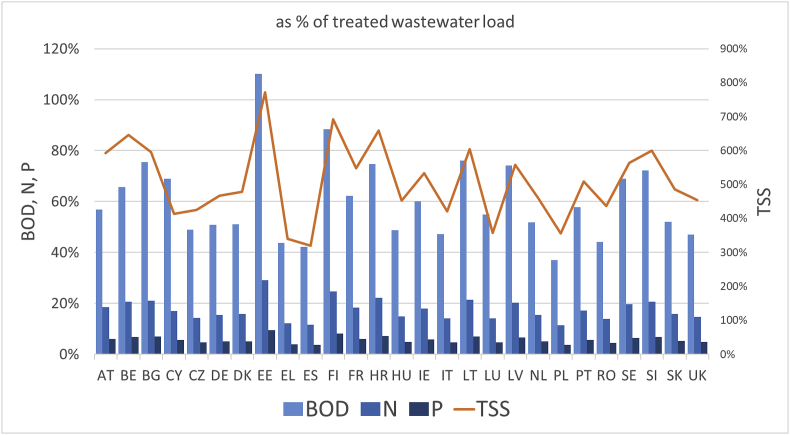
Estimated loads from urban runoff as a percentage of loads generated by treatment of urban wastewater by European standards.

It is also useful to compare the loads from urban runoff with those from treated wastewater. Fig. 2 summarizes the total loads as a percentage of loads from treated wastewater for various European Union member states. These are evaluated considering a removal efficiency of 90% for TSS and BOD_5_, and 80% for N and P, as stipulated by the European Union's Urban Wastewater Treatment Directive (UWWTD) 91/271/EEC, and the total population of each country. In order to convert the population into “population equivalents”, we use the multiplication factor 1.23 reflecting pollution loads of origins other than households (Vigiak et al., 2018). Runoff is shown to account for a significant share of the total pollution from urban areas: it represents 51% of the load of treated wastewater for BOD_5_, 15% of that of N and P, and 461% of that of TSS, about one order of magnitude higher than BOD_5_.

**Fig. 3:**
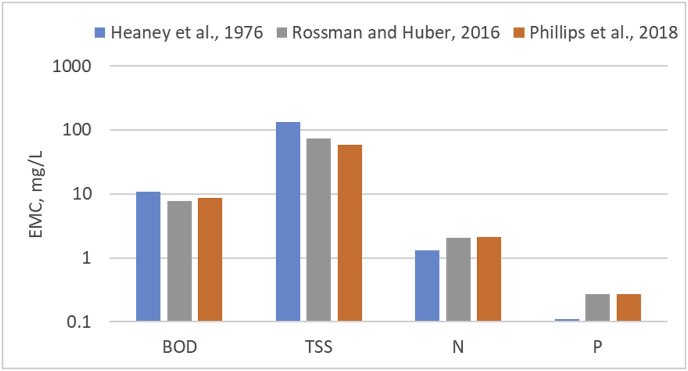
Comparison of EMC for BOD, TSS, N and P according to the model parameterization by Heaney et al. (1976), and values referece values reported by other authors.

For comparison, we refer to the EMC values summarized in Rossman and Huber (2016), based on the outcomes of the US Nationwide Urban Runoff Program (USEPA, 1983). The Authors summarize median values of EMC for residential, commercial, mixed and open/non-urban areas (Table 2). These EMC values are substantially in line with those summarized by Phillips et al. (2018), based on the work of Pitts et al. (2004) (also shown in Table 2). For N, the bulk EMC of Rossman and Huber (2016), and the median value reported by Phillips et al. (2018), refer to Total Kjeldahl Nitrogen (TKN), which is about 2/3 of total N, hence the EMC values of Table 2 are multiplied by 1.5 in the calculation. In order to apply the EMC values of Rossman and Huber (2016), in Equation (1), we compute runoff from each CLC2012 class by multiplying the annual precipitation volume falling on that category, by the degree of imperviousness from the European LUISA dataset (Lavalle et al., 2015). The product of the EMC and runoff volume is the pollution load from each CLC2012 class. The slope of the ordinary least squares (OLS) best fitting zero-intercept linear model for the total load of each pollutant as a function of the total urban runoff for each region is interpreted as a “bulk EMC”, with loads estimated both with the Heaney et al. (1976), parameterization, and with the Rossman and Huber (2016), parameterization. Fig. 3 shows a comparison of the “bulk EMC” for BOD_5_, P, N and TSS with the two approaches, as well as the median values reported for reference by Phillips et al. (2018). All values are rather consistent, with the exception of P. In this case, the values following Rossman and Huber (2016), and Phillips et al., 2018, are about 3 times as high as those following Heaney et al. (1976), and comparable with the EMC suggested by these authors for total phosphate ion (as PO_4_). The “bulk EMC” based on Heaney et al. (1976), for BOD_5_ (10.84 mg/L) is also in line with the findings of Vigiak et al. (2019), who calibrate an EMC of 11.5 mg/L for BOD_5_ using relatively recent measurements in European rivers. The bulk EMC with the other approaches remains well below 10 mg/L. All in all, in spite of having been proposed more than 40 years ago, the EMC of Heaney et al. (1976), seems comparable with more recent evidence.

**Table 2:** EMC for selected pollutants for different land use types, according to Rossman and Huber (2016), and median values reported in Phillips et al. (2018). Values in mg/L.

Land use	BOD_5_	Suspended solids	Total P	Total Kjeldahl N
Rossman and Huber (2016): Residential	10	101	0.383	1.9
Rossman and Huber (2016): Commercial	9.3	67	0.201	1.18
Rossman and Huber (2016): Mixed	7.8	69	0.263	1.29
Rossman and Huber (2016): Open – non-urban	–	70	0.121	0.54
Phillips et al. (2018)	8.6	58	0.27	1.4

The above results refer to conventional pollutants, while urban runoff may represent an even more important source of other contaminants, particularly heavy metals, polycyclic aromatic hydrocarbons (PAHs) and microplastics (Aleksander-Kwaterczak and Plenzler, 2019; Bak et al., 2019; Dikareva and Simon, 2019; Rǎdulescu et al., 2019; Sakson et al., 2018; Petrucci et al., 2014, Valtanen et al., 2015). Rügner et al. (2019), show TSS concentration to be a good predictor of PAHs. While both Rossman and Huber (2016), and Phillips et al. (2018) provide a summary of EMC also for metals and other constituents, the EMC values of the are within more than a factor 3 for Cu, 15 for Zn and 13 for Pb, indicating a higher variability. When it is not possible to describe concentrations in a more accurate way, runoff volume becomes an indicator of pollution in itself.

## 4 Conclusions

This work presents a first quantification of pollution conveyed by urban runoff at European continental scale, using a simple model based on the concept of event mean concentration (EMC). Loads of BOD_5_, TSS and nutrients from urban runoff are now comparable with those conveyed by treated wastewater in Europe, and should be considered as such in the river basin management plans implementing the European Water Framework Directive 2000/60/EC. Moreover, for certain contaminants urban runoff may represent a primary source, possibly requiring specific treatment (hence legal provisions).

The uncertainty associated with the model used in this work is known to be high, and an improved estimation requires calibrating a more sophisticated and detailed model. This in turn requires data, which are not only unavailable at present, but also unlikely to become available at any time in the future.

However, a less uncertain model is not necessary to support the policy recommendation that a reduction of urban runoff volume is key to control pollution. In the absence of specific active policies, artificial land areas (a proxy for urban surfaces) have increased in the European Union from 1990 to 2018 at a pace between 1,000 and 500 km^2^ per year, roughly 0.25–0.5% per year from an initial extent slightly below 200,000 km^2^ (https://www.eea.europa.eu/data-and-maps/indicators/land-take-3/assessment). Pollution from diffuse urban sources has arguably increased with a similar progression. Climate change is projected to cause more precipitation (hence urban runoff volume) in the North of Europe, and less in the South (Bisselink et al., 2018). However, even with less precipitation the hydrologic cycle is expected to become more impulsive, with longer dry spells and more intense storms (Heinrich and Gobiet, 2012), potentially enhancing both build-up and wash-out of pollution from urban surfaces. Therefore, climate change may worsen urban water pollution both in the North and in the South of Europe, although quantification is not possible within the scope of this assessment.

Runoff volume reduction, e.g. through urban greening, is usually expensive when considering existing drainage systems. However, it may be much cheaper if incorporated at early stages of urban planning and design. In such cases, it may even reduce the total costs of urban drainage in comparison with “grey” infrastructure, in addition to delivering other important co-benefits (e.g. Pistocchi et al., 2017), which make it an obvious no-regret option.
